# Identifying facilitators and barriers to implementing the Feverkidstool, a clinical decision tool, in the emergency department: a qualitative study in the Netherlands

**DOI:** 10.1136/bmjopen-2025-106788

**Published:** 2026-01-22

**Authors:** Sanne Vrijlandt, Erwin Ista, Ruth Kuiper, Mirjam van Veen, Anne-Marie van Wermeskerken, Fabienne Ropers, Rianne Oostenbrink

**Affiliations:** 1 Department of General Pediatrics, Erasmus MC Sophia, Rotterdam, The Netherlands; 2 Department of Neonatal and Pediatric Intensive Care, Division of Pediatric Intensive Care, Erasmus MC Sophia Childrens Hosp, Rotterdam, The Netherlands; 3 Department of Internal Medicine, Erasmus MC Universitair Medisch Centrum Rotterdam, Rotterdam, The Netherlands; 4 Department of General Pediatrics, Sophia Children’s Hospital, Rotterdam, The Netherlands; 5 Department of Pediatrics, Juliana Children’s Hospital, The Hague, The Netherlands; 6 Department of Pediatrics, Flevoziekenhuis, Almere, The Netherlands; 7 Pediatrics, Leiden Universitair Medisch Centrum, Leiden, The Netherlands

**Keywords:** Implementation Science, Clinical Decision-Making, Emergency Departments, Paediatric infectious disease & immunisation

## Abstract

**Abstract:**

**Objectives:**

This study aimed to identify determinants that hinder or facilitate implementation of the Feverkidstool, a clinical decision support tool offering a quantitative, evidence-based approach, to manage children with fever in the emergency department (ED) setting.

**Design:**

Qualitative study using semistructured interviews, analysed through directed content analysis guided by the Consolidated Framework for Implementation Research (CFIR).

**Setting:**

Secondary and tertiary paediatric emergency departments in three hospitals in the Netherlands.

**Participants:**

Eighteen potential end users of the Feverkidstool, including paediatricians and paediatric residents working in the ED and involved in the care of febrile children, participated in the study.

**Primary outcome measure:**

Determinants of Feverkidstool implementation, categorised by CFIR domains: intervention characteristics, outer setting, inner setting, characteristics of individuals and implementation process.

**Results:**

Respondents (n=18) perceived the evidence-based guidance by the Feverkidstool and its potential to reduce antibiotic use as valuable. However, concerns were raised about its applicability to critically ill children and those with comorbidities. User-friendliness was seen as a facilitator, whereas the need for C reactive protein testing and lack of integration with electronic health records were mentioned as barriers. The ability to standardise care for febrile children was considered an important benefit of using the Feverkidstool.

**Conclusion:**

Barriers and facilitators across all CFIR domains are identified. Addressing these will facilitate implementation. When effectively implemented, the Feverkidstool has the potential to improve care for children presenting with fever in the ED. This may potentially lead to a more standardised approach and reduce unnecessary antibiotic prescriptions.

**STRENGTHS AND LIMITATIONS OF THIS STUDY**

- Use of the Consolidated Framework for Implementation Research in both data collection and analysis supported a systematic and theory-informed approach.
- The purposive inclusion of participants from both academic and non-academic hospitals, with varying levels of clinical experience, ensured a broad representation of perspectives from key end users and enhanced the generalisability of the findings.
- No member checking was conducted, which may have limited the opportunity to verify interpretations.
- Potential for researcher and selection bias existed due to the use of purposive and snowball sampling. However, to reduce interviewer bias, a structured interview guide was used to promote consistency across interviews.

## Introduction

**Figure 1:**
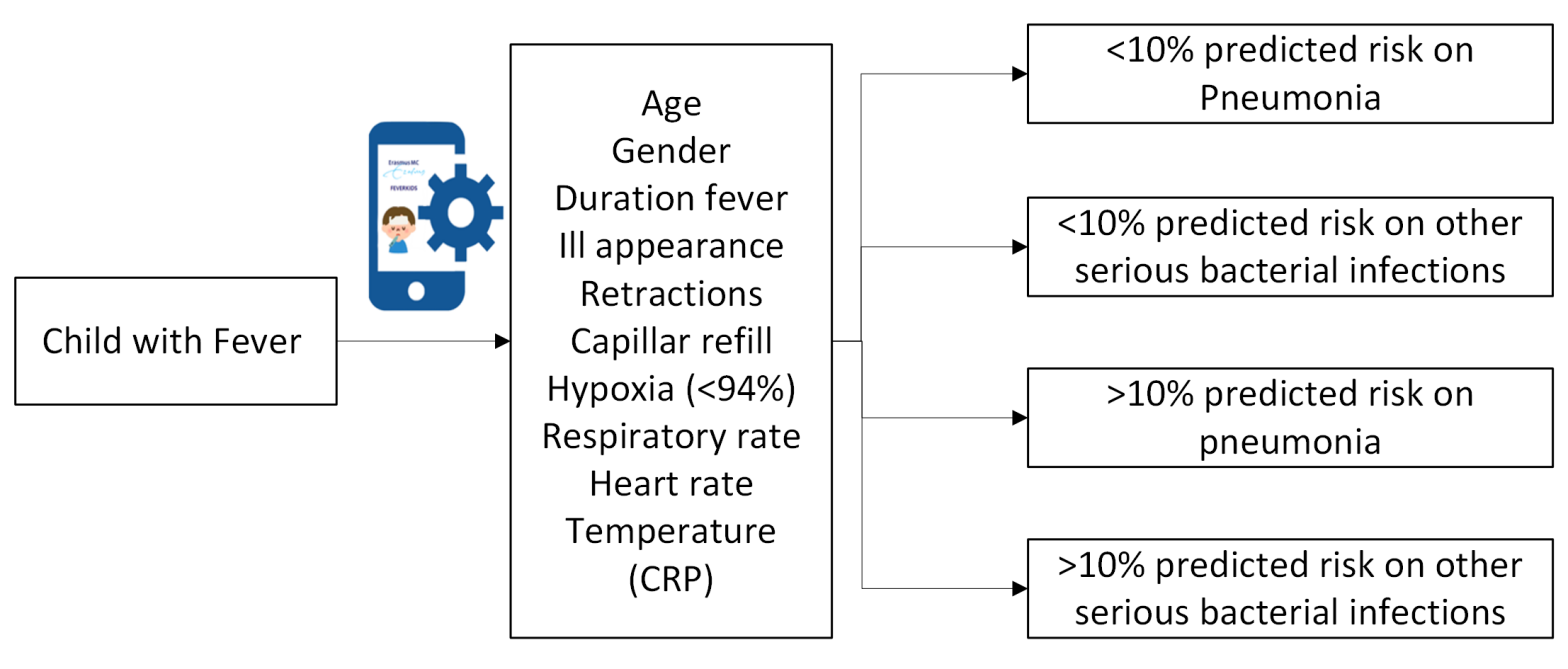
The Feverkidstool: overview of included clinical variables. CRP, C reactive protein.

 Fever is a common reason for paediatric emergency department (ED) visits, accounting for 20% of all visits,^1^ though only 5%–15% involve a serious bacterial infection (SBI).^2^ While most children suffer from self-limiting illnesses, untreated SBIs can lead to significant morbidity and even mortality. Current guidelines aim to help physicians working in paediatric emergency care to distinguish SBI from non-SBI using a list of primarily qualitative alarm symptoms; such as inconsolable crying and lethargy, leading to further diagnostics or treatment if present. In contrast, the Feverkidstool (FKT), a clinical decision tool (CDT), offers a quantitative approach by predicting individualised risks for both SBI and bacterial pneumonia based on 11 clinical variables, including C reactive protein (CRP) (figure 1).^3^ The FKT has been used in research and is externally validated in international studies, demonstrating good discriminatory ability.^4 5^ Recently, the tool has received Conformité Européenne (CE) certification to enhance broader implementation.^6^ The revised Dutch national guideline recommends incorporating the FKT, as one of the CDTs for managing paediatric fever, in daily practice.^7^

Nonetheless, the availability of an evidence-based tool and the recommendation for its use do not guarantee immediate or successful implementation.^8^ Implementation might be hindered by commonly identified implementation barriers, including infrastructure and technical difficulties, resistance to change, insufficient understanding of the technology, time constraints, high workload and legal or ethical concerns.^9^ As a CDT, the integration of the FKT faces additional challenges specific to clinical decision support systems, particularly the need to fit existing workflows and clinical contexts.^10^ The complexity of the ED environment, marked by unpredictability, high turnover and time pressure, further complicates the implementation process. A systematic review of predictive model implementation in EDs highlights the importance of early stakeholder engagement.^11^ However, most included studies were conducted postimplementation, potentially overlooking key aspects of the implementation process. Additionally, there was limited focus on clinical decision-making tools for paediatric patients, highlighting a gap in the literature regarding implementation factors for this population and its healthcare providers. Although impact analysis has been performed for the FKT,^5 12^ no research has yet been performed to specifically examine implementation of the FKT. Therefore, this study aims to obtain insights into anticipated enabling or facilitating factors for implementing the FKT in EDs, as perceived by physicians working in the ED. Understanding these factors will guide the development of an effective implementation strategy to integrate the FKT into daily ED practice.

## Methods

### Study design

We conducted a qualitative study using semistructured interviews, with key stakeholders (paediatricians and residents in paediatrics) in three Dutch hospitals. These hospitals include a large academic hospital (Leiden University Medical Center), a large teaching hospital (Haga Hospital) and a smaller regional hospital (Flevo Hospital). Qualitative data can provide rich in-depth information about the cognitions, motivations and experiences of individuals, which is well-suited for this study.^13^ Study methods and results were reported in accordance with the Consolidated Criteria for Reporting Qualitative Studies guideline (online supplemental material).^14^

### Participants

Potential end users of the FKT were recruited for the interviews. In the Netherlands, paediatric emergency medicine is not formally recognised as a subspecialty; however, all paediatricians and paediatric residents receive training in the assessment of children with emergency conditions^15^ and were, therefore, eligible for recruitment. Technical experts, though essential for implementation, were not recruited as they will not be end users and are primarily needed for tasks requiring one-time setup rather than ongoing application of the tool. For inclusion, we required a minimum of 6 months of experience as a paediatrician or paediatric resident, including clinical work in the ED. Individuals involved in the scientific development of the tool were excluded from participation. Participants were recruited through snowball sampling and selected purposely to ensure a varied group of end users with respect to setting and experience. Selected professionals were invited by e-mail, with up to three follow-up reminders sent if necessary. The aim was to interview a minimum of 12 professionals, as suggested by Francis *et al*.^16^ Inclusion stopped once no novel ideas or perspectives emerged during three consecutive interviews. This data saturation ensured a comprehensive and complete representation of viewpoints.^16^

### Patient and public involvement

Patients were not involved in this study as this research focuses on the end users of the FKT.

### The CFIR

**Figure 2:**
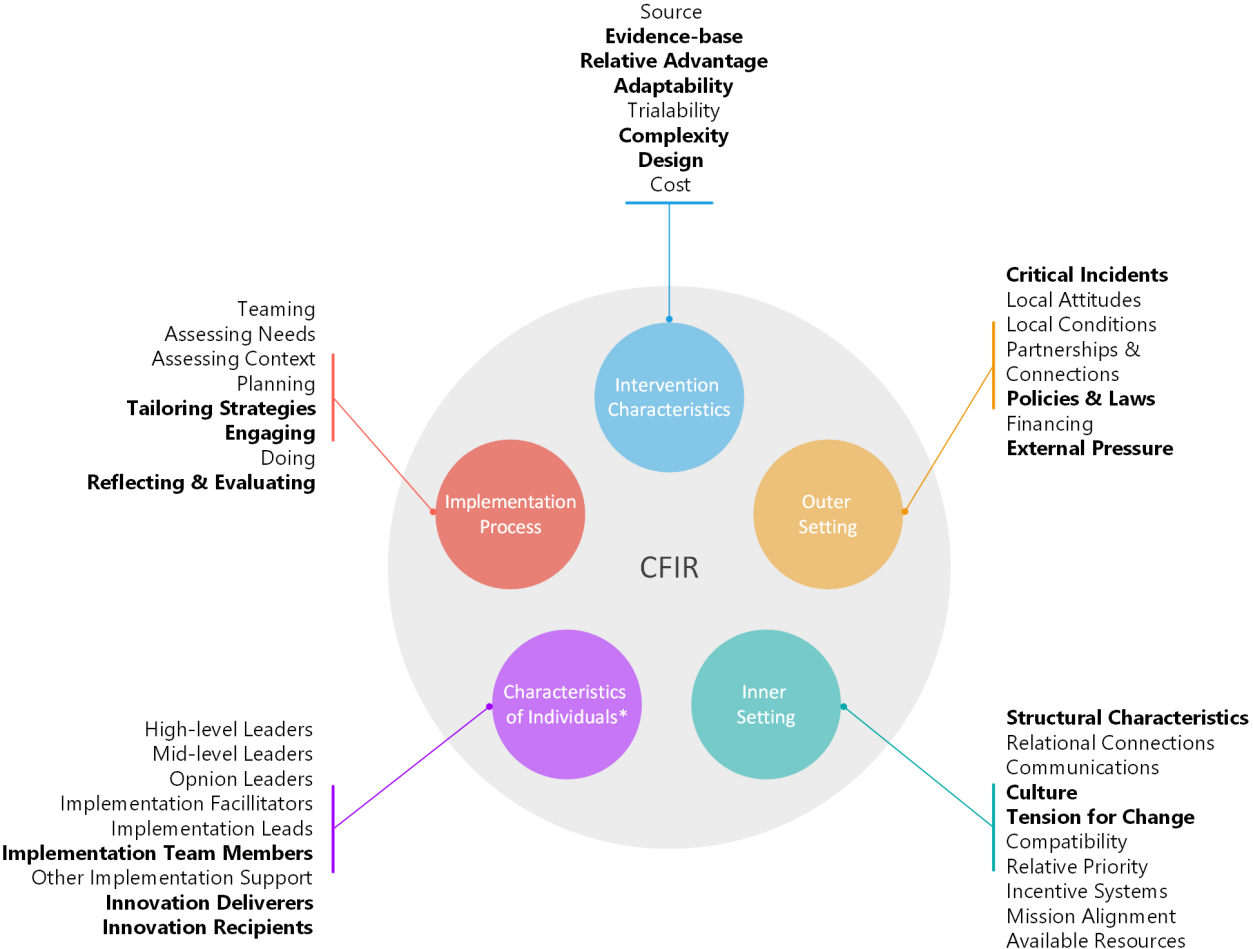
Consolidated Framework for Implementation Research.^17^ CFIR, Consolidated Framework for Implementation Research. *All constructs in this domain can be further divided into need, capability, opportunity and motivation. Constructs used in this study are in bold.

The Consolidated Framework for Implementation Research (CFIR) was used to structure the development and analysis of the interviews.^17^ The CFIR is a widely used theoretical framework combining five domains influencing the implementation of evidence-based innovations (figure 2). The framework can be tailored to the intervention and setting. The framework includes five domains: the innovation, the inner setting, the outer setting, the individuals involved and the implementation process. Together, these domains encompass 48 constructs.^17^ By systematically examining the domains (using the constructs), we could identify elements that influenced the FKT’s implementation.

### Data collection

The semistructured interview guide (online supplemental material) was developed based on the five CFIR domains. The guide was reviewed by an implementation expert (author EI) and piloted three times before being finalised with the senior author (RO). Participants received an information and consent form prior to the interviews, delineating the interview’s scope. Interviews were held via digital meetings (Microsoft Teams) by two female researchers; the first (SV, MD, PhD student) and third author (RK, BSc, Medical student), both trained in conducting interviews. In instances where the interviewer had prior acquaintance with the participant, the interview was conducted by the other researcher. Interviews were conducted on a one-on-one basis, and no follow-up interviews were required. Prior to starting the interview, the goal of the interview and the FKT was explained.

### Analysis

Interviews were audio-recorded and transcribed for analysis manually. To avoid interrupting the flow of conversation, no field notes were taken during the interviews. First, the whole dataset, including all transcripts, was read by two researchers to familiarise themselves with the data. Next, directed content analysis, a method suited for extending an existing theory or framework, was used to analyse the interviews based on the CFIR framework.^18^ The initial coding phase involved two researchers independently coding the first six interviews based on the CFIR codebook. Discrepancies in coding between interpreters were discussed, and a consensus was reached on the coding approach. Subsequent interviews were coded by one of the researchers and then evaluated by the other, with discussion and potential revision if necessary. After, the codebook was discussed with the last author and further refined by categorising the codes into the CFIR constructs. This subdivision was discussed with our implementation scientist (EI). Additionally, the codes were divided into facilitators, barriers or both. Disagreements were resolved through discussion. No feedback on the findings was provided by participants.

## Results

A total of 23 physicians working at the paediatric department were invited for an interview. Five did not respond or were not interested, resulting in 18 interviewees: eight paediatricians, five residents not in training, four residents in training to become a paediatrician and one resident in training to become a doctor in international healthcare (table 1). The majority (15) were female. Work experience at the paediatric ED ranged from 6 months to 20 years. The interviews lasted between 14 and 45 min (median: 24 min; IQR 21–30).

**Table 1:** General characteristics of participants

Characteristics (n=18)	n (%)
Gender	
Female	15 (83.3)
Hospital	
Academic medical centre	7 (38.9)
Teaching hospital	5 (27.8)
General hospital	6 (33.3)
Occupation	
Paediatrician	8 (44.4)
Resident not in training	5 (27.8)
Resident in training	5 (27.8)
Paediatric medical practice experience (years)	
< 2	6 (33.3)
2–<5	1 (5.6)
5–<10	5 (27.8)
10+	6 (33.3)
Paediatric emergency department experience (years)	
<2	7 (38.9)
2–<5	0 (0.0)
5–<10	6 (33.3)
10+	5 (27.8)
Familiar with Feverkidstool	
Yes	8 (44.4)

Based on the interviews, codes were identified within 21 constructs, out of the 48 constructs in the CFIR guide during the interviews (marked in bold in figure 2). Of these constructs, nine included only facilitators, three included only barriers and eight were both. Table 2 provides an overview of the barriers and facilitators, including illustrative quotes, according to the CFIR domains and constructs.

**Table 2:** Barriers and facilitators for implementing the Feverkidstool within the CFIR Framework

CFIR domain	Construct	Description facilitator (F)/barrier (B)	Quote examples within construct
(1) Innovation	Evidence-based	Scientific evidence provided by different studies (F)*	‘’If you hear those results, then I think you can truly make a difference with them.’’ (A2)
	Relative advantage	Physicians with limited clinical experience (F)* Cases of uncertainty (F)* Efficient healthcare processes (F)* Increased objectivity/standardised care (F)* Provides guidance (F)* Reduced use of antibiotics (F)* Confidence in own clinical judgement (B)* Full reliance on the tool (B)* No added value in acute ill children (B) No added value in children with a clearly defined diagnosis (B)*	‘I think that when deciding whether or not to give antibiotics, gut feelings very often play a role, and you can perhaps make this a bit more objective with this.’ (B4) ‘If a child comes to the ED septic, then there is no doubt about starting treatment.’ (C5)
	Adaptability	It does not take comorbidities into account (B)	‘But our population is different; This is not the standard population, and therefore its use will be limited.’ (B3)
	Complexity	Easy to use (short and clear questions) (F)* CRP needed to use full version (B)	‘What I personally find the most challenging is the inclusion of CRP.’ (B2)
	Design	Possibility to use it via smartphone or computer (F)* Not available as app (B)* Not available or link with EPD (B)*	‘That it is good that it is actually implemented in an EPD, so you don't have to work with separate apps again.’ (B6)
(2) Outer setting	Critical incidents	Afraid of consequences when using the tool (F)*	‘ I would probably feel the need to thoroughly explain in the record why I might deviate from it.’ (B4)
	Policies and Laws	Incorporation in the national guideline (F)* Satisfaction with the current guideline (F/B)	‘If the Feverkidstool were also included in the guideline for fever without focus, I think this would be appropriate.’ (B6)
	External pressure	Antibiotic resistance as growing problem (F)	‘I believe that a lot of antibiotics are currently being given to children who don't actually need them.’ (B5)
(3) Inner setting	Structural characteristics	Final decisions differ per supervisor (F)* No extra workload for nurses (F)* High workload/limited time (B)	‘I often notice that if you sometimes see the same patient two days in a row with a different supervisor, you suddenly have to explain a completely different argument to the same people.’ (C3)
	Culture	Openness to using the tool (F/B)*	‘I can imagine that initially, deviations will be frequent, but as time goes on and the tool demonstrates its usefulness, deviations will become less common.’ (B3)
	Tension for change	There is not much debate about the current situation (B)*	‘ I don't feel that things are necessarily going very wrong right now.’ (B4)
(4) Individuals	Implementation team members	Universal adoption of the tool (F)* Involve nurses (F)*	‘No. It is, I do think that it is nice that the nurses are also aware of it.’ (B7)
	Innovation deliverers	Changing and starting residents (F) Curiosity to use the tool (F)* In uncertain cases (F)* Possibility to test your own clinical judgement (F)* As physician you are the one end responsible for a decision (B) Change is naturally challenging (B) Only available to use as physician (B)	‘I believe that older doctors might prefer to continue doing things their own way and may be less influenced by the results of such a tool.’ (C3) ‘But, I find it interesting to test myself with it.’ (B4)
	Innovation recipients	The tool could lead to less invasive diagnostic procedures (F) The tool could support explaining the decision for a treatment procedure (F)* Parents like to hear a doctors reasoning rather than that of a computer-based tool (B)	‘I do think that in cases where there are multiple possibilities, so to speak, where you lean more towards shared decision-making, it is good to include this as well.’ (A1)
(5) Implementation process	Tailoring strategies	Apply different implementation methods (F)*	‘That a presentation is sent, so that we also have it on our V-drive, … A poster in the doctors’ room would certainly help as well.’ (A5)
	Engaging	Involve key users with affinity for the subject (F)*	‘I think you need a driving force in each center.’ (B3)
	Reflecting and evaluating	Evaluate and update the tool (F)*	‘So easily accessible, and I also think evaluating and updating the tool is important, yes.’ (C4)

* Indicates facilitators or barriers mentioned five times or more.

CFIR, Consolidated Framework for Implementation Research; CRP, C reactive protein; EPD, Electronic Patient Dossier.

Respondents in the last column are identified by letter–number combinations with letters representing the hospitals (A, B, C), and numbers identifying individual respondents within each hospital.

### Intervention characteristics

Available scientific evidence was important for participants as mentioned by all of them, the extensive research and promising results would facilitate the tool usage. The possibility to reduce antibiotic prescription was seen as a relative advantage of the tool. Additionally, most participants valued the tool’s ability to support more objective decision-making, which could lead to more consistent and evidence-based clinical decisions. However, the use of the tool would not change practice regarding starting antibiotic treatment to critically ill patients and patients with a clear focus of infection (reported by >5 respondents). Also, some would not use the tool for children with comorbidities as antibiotics are often started more quickly in this group due to a perceived higher risk of serious infection (reported by <5 respondents). While participants found the tool user-friendly, they suggested improvements primarily related to the login procedure, like an application or electronic health record (EHR) integration. Another concern raised was the requirement for CRP testing as laboratory testing is not part of the routine workup for children presenting to the ED with fever, potentially limiting full adoption.

### Inner setting domain

Participants acknowledged that inner settings vary between hospitals, particularly due to differences in patient populations. They believed the tool may be more applicable in cases involving less severely ill children, where uncertainty and differing opinions regarding management are greater, potentially leading to differences in uptake across hospital settings. Paediatric residents additionally noted that treatment approaches varied between supervising paediatricians within hospitals (reported by >5 respondents). Nurses not measuring vital signs, potentially leading to missing data for the tool, were not seen as a barrier by participants, as this was mentioned to occur infrequently. Involving nurses in the implementation process was deemed essential, primarily to help remind paediatricians use the tool. Although there was no strong pressure to change, participants acknowledged that antibiotic overuse is a significant concern (reported by <5 respondents). Most participants highlighted the importance of ensuring the tool is used consistently by all staff, noting that as the tool demonstrates its effectiveness, its adoption is likely to increase throughout the department.

### Outer setting

Participants expressed no concerns for critical incidents, like patient claims, when deviating from the tool’s recommendation. However, they did emphasise the importance of comprehensive documentation. Additionally, integrating the tool into national guidelines and working with the Dutch Association of Pediatrics is cited as important for its usage, promoting more objective and safer paediatric care.

### Individual domain

Almost all users indicated that their likelihood of adopting the tool would increase if their colleagues also used it. Participants saw potential benefits in involving nurses, who should at least be familiar with the tool to encourage its use by doctors and ask about it when needed (reported by >5 respondents). There was also interest in expanding the tool to general practitioners as they mostly perform the initial assessment. Participants recognised their responsibility in patient care and acknowledged the importance of using clinical intuition in conjunction with the tool, rather than relying on the tool alone. Interviews revealed a discrepancy between less and more experienced doctors. All experienced doctors (paediatricians and residents in training) tend to rely more confidently on their own intuition but believe the tool is valuable for less experienced colleagues, who mentioned relying on their own intuition less frequently. Participants, particularly paediatricians, saw the tool as useful for testing their clinical judgement. Some physicians mentioned that using it to explain decisions to patients could be valuable, but this was not considered useful by all.

### Implementation domain

Various implementation strategies were mentioned by all participants; however, most emphasised the importance of a presentation to motivate and inform users. They indicated that this presentation should combine scientific evidence from validation studies, practical demonstrations and detailed usage instructions. Many also recommended appointing a key stakeholder at each centre to lead the implementation. Finally, participants proposed that regularly evaluating and updating the tool, through data analysis and case discussions, and sharing these findings would further enhance its use.

## Discussion

This qualitative study revealed facilitators and barriers, across all five domains of the CFIR as reported by end users. Key barriers found within the innovation domain included the expected limited added value for patients with (1) comorbidities, (2) a clear diagnosis and (3) in critically ill children. However, an important facilitator within this domain was also mentioned; the tool’s ability to provide clearer guidance, resulting in more standardised advice. Besides providing advice, the FKT also contributes to a standardised evaluation of febrile children, which may be more transferable than clinicians’ intuition across different healthcare providers during supervision or shift transitions.

Important factors influencing the adoption of the FKT in the ED appeared to be the evidence and the perceived usefulness, also noted in previous studies.^9 10^ The FKT could help prevent unnecessary diagnostic procedures and reduce the frequent use of antibiotics in cases of uncertainty (mentioned by almost all participants).^5^ One of the barriers mentioned most often was the user’s opinion that the current practice was acceptable. This perception may be due to the relatively infrequent occurrence of SBIs and a very low incidence of invasive bacterial infections with fast fatal course if missed.^19^ Because such cases are uncommon, the potential harm of undertreatment may go unnoticed by individual providers. Side effects of unnecessary antibiotic treatment in febrile children with self-limiting viral infections may not be recognised, as the impact becomes evident in the long term. Also, the preference of action over inaction, uncertainty, the lack of continuity of care by the same physician and limited follow-up on treatment outcomes strengthens not identifying the need for practice change.

The FKT was perceived as less beneficial in patients with complex underlying medical conditions align with the literature, where barriers were found due to a tool’s inflexibility in adapting to specific contexts, such as clinical contexts and specific illnesses.^10^ This was particularly mentioned by physicians in academic hospitals. However, an external validation study involving febrile immunocompromised children demonstrated the tool’s potential to reduce unnecessary investigations and antibiotic use in high-risk groups.^20^ The FKT can be implemented across all hospitals, although its primary benefit is expected in children triaged with lower urgency.^21^ Additionally, it is crucial to further assess its usability and effectiveness in routine practice through postmarket surveillance. If deemed necessary, adaptations to better meet the needs of patients with comorbidities could be explored.

Barriers such as high workload and limited time, due to extra administrative tasks, were concerns, this is also noted in several studies.^22^ To address this issue, integrating the FKT into clinical pathways is essential. Ultimately, embedding the tool within EHRs would serve as reminder for clinicians and facilitate optimal integration, potentially resulting in increased usage and decreased extra administration time.^19 23^

Incorporating the tool into existing protocols was also mentioned as facilitator. While protocols are commonly employed in the ED, adherence can vary significantly.^24^ This variability implies that simply integrating the tool into protocols or guidelines may not be sufficient to ensure its widespread use. The potential threat to professional autonomy, a barrier found in previous studies,^22 25 26^ was not mentioned in this study. Most physicians felt confident in negotiating the tool’s advice, seeing it as an aid rather than a replacement for diagnostic reasoning. In addition, the FKT provides a single-point assessment with immediate advice, whereas infectious conditions can evolve over time, progressing from subtle early signs to clear management indications. End users particularly valued the immediate advice of the tool in less clear, early phase presentations with highest diagnostic uncertainty (see quotations in the construct ‘relative advantage’ in table 2). This aligns with the way the FKT, and other CDSTs, should be used by complementing rather than replacing physicians’ clinical judgement, and that recommendations should always be interpreted within the broader clinical context of the individual patient. The FKT has been validated for single-moment assessment only, and its use beyond this context has not been formally evaluated.

Using multiple implementation strategies was mentioned as a facilitator. All participants expressed a desire for training to familiarise themselves with the FKT, both individually and as a group. As the FKT is perceived as easy to use, training requirements are expected to be minimal. The suggestion to appoint a local key user in every organisation to answer questions and remind everyone to use the tool during the implementation process is also mentioned in the existing literature.^27^ Also, including nurses in the implementation process is essential to support full uptake. Because nurses perform the initial assessment, they are well-positioned to identify eligible patients and initiate early use of the tool. Their active involvement, therefore, facilitates integration of the tool into routine practice in a more coordinated, team-based manner.

### Strengths and limitations

A strength of this study was the inclusion of participants with diverse levels of work experience from both academic and non-academic hospitals. Perspectives from both paediatricians and residents within the paediatric department were incorporated, aiming to achieve an equal representation of these groups. Also, utilising the CFIR in both data collection and analysis facilitated a comprehensive examination of barriers and facilitators impacting implementation.

As a limitation, member checking, which could have enhanced the accuracy of findings, was not used. This decision was primarily due to reduce the burden on participants. Next, there was the issue of potential researcher bias. However, this was minimised by the interviews being guided by a clear interview guide. Finally, selection bias might have occurred as participants were included based on purposive and snowball sampling.

### Further research

The results of this study will be used for a tailored implementation strategy for the FKT at the ED. The CFIR and the Expert Recommendations for Implementing Change matching tool will be used to start building this strategy.^28^ Also, when implementing the FKT internationally, stakeholders from countries with different healthcare systems should be questioned (eg, primary vs secondary care–led systems) should be consulted to ensure applicability across diverse settings and to identify context-specific barriers and drivers. Next, as implementation progresses, further research should focus on evaluating the implementation phase, including the actual use of the FKT, its usability and the cost-effectiveness and clinical-effectiveness. After, a postimplementation study can be conducted to evaluate its impact on clinical outcomes in a real clinical setting. Such evaluation is essential, as the added value of CDTs can only be determined when they are used in routine practice and assessed in relation to real-world clinical decision-making.

In this study, we identified key factors influencing the implementation of the FKT in the ED. Facilitators included its ability to provide an objective approach and its usefulness in cases of diagnostic uncertainty. However, barriers such as the limited generalisability of the FKT to severely ill children and those with comorbidity and its integration into existing workflows, particularly in the complex ED setting, were noted. Findings indicate that key actionable priorities for implementation include integrating the tool into EHR and providing evidence-based education should be central to the implementation strategy. Furthermore, this study highlights the necessity of identifying appropriate implementation strategies to ensure wide adoption, especially in complex settings such as the ED. Next steps involve pilot testing and evaluating usability, adoption and clinical impact, acknowledging that the FKT is intended to support clinical decision-making by complementing physicians’ clinical judgement within the broader clinical context. Additionally, exploring implementation in different international healthcare systems will be important to adapt strategies to local contexts. Ultimately, implementation could lead to a more standardised approach and a reduction in unnecessary antibiotic prescriptions for children with fever in the ED.

## Supplementary material

10.1136/bmjopen-2025-106788 online supplemental file 1

## Data Availability

No data are available.
