# Supplementary material for: Identifying facilitators and barriers to implementing the Feverkidstool, a clinical decision tool, in the emergency department: a qualitative study in the Netherlands
Source: BMJ Open. 2026 Jan 22;16(1):e106788. doi: 10.1136/bmjopen-2025-106788 (PMC12829391; doi:10.1136/bmjopen-2025-106788)
Supplement: online supplemental file 1 [file bmjopen-16-1-s001.docx]

**Supplementary material**

**Supplementary material 1.**
**Consolidated criteria for reporting qualitative studies (COREQ): 32-item checklist**

| No. Item | Guide questions/description | Reported on Page # |
| --- | --- | --- |
| Domain 1: Research team and reﬂexivity |  |  |
| *Personal Characteristics* |  |  |
| 1. Inter viewer/facilitator | Which author/s conducted the inter view or focus group? | 9 |
| 2. Credentials | What were the researcher’s credentials? E.g. PhD, MD | 9 |
| 3. Occupation | What was their occupation at the time of the study? | 9 |
| 4. Gender | Was the researcher male or female? | 9 |
| 5. Experience and training | What experience or training did the researcher have? | 9 |
| *Relationship with participants* |  |  |
| 6. Relationship established | Was a relationship established prior to study commencement? | 9 |
| 7. Participant knowledge of the interviewer | What did the participants know about the researcher? e.g. personal goals, reasons for doing the research | 9 |
| 8. Interviewer characteristics | What characteristics were reported about the inter viewer/facilitator? e.g. Bias, assumptions, reasons and interests in the research topic | 9 |

| Domain 2: study design |  |  |
| --- | --- | --- |
| *Theoretical framework* |  |  |
| 9. Methodological orientation and Theory | What methodological orientation was stated to underpin the study? e.g. grounded theory, discourse analysis, ethnography, phenomenology, content analysis | 8 - 10 |
| *Participant selection* |  |  |
| 10. Sampling | How were participants selected? e.g. purposive, convenience, consecutive, snowball | 8 |
| 11. Method of approach | How were participants approached? e.g. face-to-face, telephone, mail, email | 8 |
| 12. Sample size | How many participants were in the study? | 8,10 |
| 13. Non-participation | How many people refused to participate or dropped out? Reasons? | 10 |
| *Setting* |  |  |
| 14. Setting of data collection | Where was the data collected? e.g. home, clinic, workplace | 9 |
| 15. Presence of non-participants | Was anyone else present besides the participants and researchers? | 9 |
| 16. Description of sample | What are the important characteristics of the sample? e.g. demographic data, date | 10 |
| *Data collection* |  |  |
| 17. Interview guide | Were questions, prompts, guides provided by the authors? Was it pilot tested? | 9 |
| 18. Repeat interviews | Were repeat inter views carried out? If yes, how many? | 9 |
| 19. Audio/visual recording | Did the research use audio or visual recording to collect the data? | 9 |
| 20. Field notes | Were ﬁeld notes made during and/or after the interview or focus group? | 9 |
| 21. Duration | What was the duration of the inter views or focus group? | 10 |
| 22. Data saturation | Was data saturation discussed? | 8 |
| 23. Transcripts returned | Were transcripts returned to participants for comment and/or correction? | 19 |
| Domain 3: analysis and ﬁndings |  |  |
| *Data analysis* |  |  |
| 24. Number of data coders | How many data coders coded the data? | 9, 10 |
| 25. Description of the coding tree | Did authors provide a description of the coding tree? | 9, 10 |
| 26. Derivation of themes | Were themes identiﬁed in advance or derived from the data? | 9, 10 |
| 27. Software | What software, if applicable, was used to manage the data? | NA |
| 28. Participant checking | Did participants provide feedback on the ﬁndings? | 10 |
| *Reporting* |  |  |
| 29. Quotations presented | Were participant quotations presented to illustrate the themes/ﬁndings? Was each quotation identiﬁed? e.g. participant number | 14, 15 |
| 30. Data and ﬁndings consistent | Was there consistency between the data presented and the ﬁndings? | 17 - 20 |
| 31. Clarity of major themes | Were major themes clearly presented in the ﬁndings? | 11 - 13 |
| 32. Clarity of minor themes | Is there a description of diverse cases or discussion of minor themes? | 11 - 20 |

**Supplementary material 2.**
 **Interview guide semi structured interviews**

**Introducing questions:**

1. What is your current role?
2. In which hospital are you currently working?
3. How many years of experience do you have in Pediatric Medicine?
4. How long have you been seeing children in the Emergency Department?
5. Were you aware of the Feverkidstool before this interview?

**Main questions (*per domain*):**
 Intervention characteristics

1. In research settings, the FKT showed antibiotic reduction in the low-risk group and less therapy failure in the high-risk group. How do you view these findings, what do you think are the implications of using the FKT?
2. Would using the FKT add to your current practices? Why of why not?
   1. Would you base or adjust your policy on the risk calculations from the FKT? Why of why not?
   2. Possible extra question: how do you think about the safety?
3. The Feverkidstool is currently available as a website where the different parameters need to be manually entered. The website can be accessed on both computers and mobile phones. What is your opinion on the ease of use of the Feverkidstool in this format?
   1. How could this be improved?
4. In your opinion, are any adjustments needed to the FKT so that it can start being used?
   1. If so, what?
   2. Where do you think is the best place to make the FKT available?

Individuals

1. The FKT aims for more standardised care in the treatment of children with fever. How do you think the FKT will contribute to achieving this goal?
2. To what extent do you think it is important that your colleagues use the tool?
   1. Does their usage influence your decision to use the tool?
3. Would you inform the patient about the use of the FKT? If yes, in what way? If not, why not?
   1. Do you believe that using the FKT has an impact on patients? If yes, what kind of impact do you think it has?

Inner setting

1. Is there a need for the FKT within your department? Why or why not?
   1. What measures do you think are necessary to raise awareness about the FKT within the medical profession?
2. Do you believe it’s crucial to change the current approach to managing children with fever in the ED? And if so, do you see the FKT as a good solution for this?
3. How do you envision integrating the FKT into current clinical practice?
   1. When would you consider utilising the Feverkidstool?
4. Do you perceive nurse collaboration as a potential barrier to implementing the FKT? If so, what are the reasons behind this perception, and what strategies could be implemented to address it?
   1. Are vital signs consistently recorded and documented?
5. Do you have concerns about the consequences of not adhering to the advice provided by the FKT if it were to be implemented? If yes, what specific consequences do you fear?
   1. Can you identify any reasons or situation that might lead to not using the tool?
   2. If needed: For instance, due to being busy, not having access to a computer, or because it’s not mentioned in the guidelines?

**Closing questions:**

1. Can you think of any other factors that might influence whether the intervention would be successful or not in your organisation?
   1. Could factors such as time constraints, uncertainty diagnosis, or reduced availability of diagnostic tools impact the use of the intervention?
2. Do you have any additional ideas that could enhance the implementation process and promote greater utilisation of the tool?
3. Do you have any other questions or comments left?
4. Do you know any other people who might want to participate in the study?
