# Supplementary material for: Identifying facilitators and barriers to implementing the Feverkidstool, a clinical decision tool, in the emergency department: a qualitative study in the Netherlands
Source: BMJ Open. 2026 Jan 22;16(1):e106788. doi: 10.1136/bmjopen-2025-106788 (PMC12829391; doi:10.1136/bmjopen-2025-106788)
Supplement: Supplementary file 2 [file bmjopen-2025-106788.reviewer_comments.pdf]

PEER REVIEW HISTORY

BMJ Open publishes all reviews undertaken for accepted manuscripts. Reviewers are asked to complete a checklist review form and are provided with free text boxes to elaborate on their assessment. These free text comments are reproduced below.

ARTICLE DETAILS

Title (Provisional)

Identifying facilitators and barriers to implementing the Feverkidstool, a clinical decision tool, in the Emergency Department: a qualitative study in the Netherlands.

Authors

Vrijlandt, Sanne; Ista, Erwin; Kuiper, Ruth; van Veen, Mirjam; van Wermeskerken, Anne-Marie; Ropers, Fabienne; Oostenbrink, Rianne

---

VERSION 1 - REVIEW

**Reviewer**	**1**
**Name**	**Rosenthal, Jennifer**
**Affiliation**	**UC Davis**
**Date**	**02-Sep-2025**
**COI**	**None**

This paper is a descriptive qualitative study identifying the barriers and facilitators to the implementation of the Feverkidstool, a clinical decision support tool used in the care of febrile children. This topic is important to readers of BMJ Open because it focuses on the implementation of an evidence-based tool designed to help standardize care and reduce antibiotic use, contributing to public health goals in addressing antibiotic resistance. This paper addresses a critical gap in the literature examining clinical care tool implementation factors in pediatric emergent care. The paper is well-written and thoroughly examined the providers' perspective on the applicability of the Feverkidstool. Various sections of the paper could use some clarification or editing. Detailed comments are provided below.

Abstract:

• It is not readily apparent what the Feverkidstool is for in the abstract. A short descriptor added to the objectives can help clarify.

• Consider presenting the Results section by CFIR domains.

• Conclusion: Be careful to not overstate the implications of this present study in the last two sentences.

Introduction:

• In the first paragraph, the Feverkidstool is briefly described ("… 11 clinical variables, including C-reactive protein.") As written, I thought other lab values would be included in the tool [in addition to CRP]. However, upon looking up Reference #3, I learned that Feverkidstool does not include any other lab values, but does include history of fever duration, vitals, and exam findings. To provide readers with more thorough understanding of Feverkidstool, consider adding a table/figure or more details to the text.

• Thorough Introduction with strong rationale supporting the need for this present study. Study does a good job at connecting the evidence with the need.

• It is unclear what research, if any, has explored implementation of Feverkidstool. Consider explicitly stating if such research has been conducted on specifically the Feverkidstool.

Methods:

• Did the inclusion criteria require a certain amount of experience in the ED (what if a resident has not rotated through the ED yet)? The paper nicely explains that pediatric emergency care is not a subspecialty; however, it remains unclear what other settings, if any, these pediatricians work (i.e., outpatient, inpatient.

• Analysis: Please provide the reason, if any, for not taking field notes.

• Was this study reviewed/approved by an ethics board?

Results:

• In general, many of the descriptions of the findings lacked depth. Statements were made about the constructs that lacked explanations for "why" and "how." For example, Intervention characteristics: Did any of the participants elaborate more on why CRP testing would hinder adoption? Or why they would not use the tool for children with comorbidities? (page 13) Another example, Implementation domain: What scientific evidence and data analysis did participants want to have (e.g., clinical outcomes data?)? (page 15)

• It would be helpful to include information throughout the Results section to clarify how common (or ubiquitous) a perception was. Also including details on whether perceptions were largely expressed by a certain provider type (which is provided in the Individual domain but not others).

• Including any notable deviants, if any, would be useful.

• Individual domain: The perspectives of more experienced doctors [about the less experienced doctors] is provided. What were the perspectives of the less experienced doctors? Were they more willing to adopt/rely on the tool? (page 14)

Table 2:

• Please clarify what the labels are in the last column. Does the "F" for "F2" have any significance? (Similarly, "L" for "L4"? "H" for "H5"?)

Discussion:

• Regarding the first sentence, is this a general implementation strategy or for a specific ED? (page 15)

• (page 15, lines 40-43) Please provide a citation, if any, supporting the statement about the tool precenting unnecessary diagnostic procedures and antibiotics.

• Paragraphs 2-4 could be rearranged to group together similar concepts (e.g. barriers related to antibiotics and bacterial infections, barriers related to comorbidities, logistical and professional barriers).

• There was a note about including nurses in implementation in the results. Given this study emphasizes the need to include key stakeholders in the implementation phase, it would be important to mention again/expand on this finding either in the discussion or in further research about incorporating nurses as key stakeholders.

Reviewer **2**

Name **Keijzers, Gerben**

Affiliation **Gold Coast Hospital and Health Service, School of Medicine**

Date **04-Sep-2025**

COI **None**

This is well conducted qualitative study assessing barriers and enablers of a clinical decision aid to determine SBI in Dutch EDs.

I only have minor comments:

1- I cannot see any ethics statement in the manuscript. I would expect particpants to provide informed consent to their interviews.

2- The Feverkidstool ideally should be explained more. On reviewing the tool, it does not have much better accuracy than existing tools or clinical gestalt. Suggest to explain the tool in the methods and acknowledge its limitations in the discussion

3- The discussion is well balanced - and indeed some of the respondents comments that any clinical decision tool should aid Gestalt rather than replace it remains important.

I would encourage the authors to read this lengthy evidence based blog post on clinical decision tools, essentially highlighting the need for it to be compared to Clinical Gestalt. This could take an additional paragraph in the discussion. See: https://first10em.com/clinical-decision-rules/

Reviewer **3**

**Name**	**Kaya, Murtaza**
**Affiliation Department**	**Kutahya Saglik Bilimleri Universitesi, Emergency**
**Date**	**17-Sep-2025**
**COI**	**None**

Dear Editor,

Thank you for the opportunity to review this manuscript. The article entitled “Identifying facilitators and barriers to implementing the Feverkidstool, a clinical decision tool, in the Emergency Department: a qualitative study” makes a valuable contribution to understanding the implementation of evidence-based clinical decision support tools in pediatric emergency care. The study is systematically designed using the CFIR framework and provides an in-depth analysis of pediatricians’ and residents’ perspectives, identifying key facilitators and barriers to implementation. The findings are clinically relevant, particularly for standardizing the assessment of febrile children and reducing unnecessary antibiotic use.

Abstract

The abstract is clear and logically structured, describing the objective, design, setting, participants, and main findings. Mentioning the CFIR framework adds methodological strength, and both facilitators and barriers are summarized.

Suggestions:

• Specify the most critical facilitator and barrier (e.g., EHR integration as a key barrier).

• Strengthen the conclusion by highlighting the potential clinical impact, such as expected antibiotic reduction.

• Include the number of participants (n=18) in the results for context.

Introduction

The introduction provides a strong rationale, describing the burden of febrile presentations and the limitations of current guideline-based approaches. The Feverkidstool’s evidence base, CE certification, and recommendation in the Dutch national guideline establish the importance of the topic.

Suggestions:

• End with a clear research question, e.g., “This study aims to systematically identify the determinants that hinder or facilitate the implementation of the Feverkidstool in pediatric EDs, guided by the CFIR framework.”

• Briefly justify why qualitative methodology is appropriate for exploring implementation challenges.

Methods

The methods are rigorous and reported according to COREQ guidelines. The use of semi-structured interviews and CFIR-based directed content analysis is highly suitable for this study aim. Inclusion of diverse hospitals and purposive sampling until saturation adds strength.

Suggestions:

• Specify the software used for data management/coding (if any) to enhance reproducibility.

• Provide the mean or median interview duration in addition to the range.

• Discuss how participants' prior familiarity with the Feverkidstool was handled analytically, as this may influence perceptions.

Results

Findings are well-structured by CFIR domains, and illustrative quotes enrich the analysis. Table 2 is comprehensive and clearly links facilitators and barriers.

Suggestions:

• Add a brief summary paragraph or figure highlighting the top three facilitators and top three barriers for quick reference.

• Where feasible, quantify themes (e.g., "12/18 participants mentioned this barrier") to indicate relative importance.

• Ensure that interpretive comments and implementation solutions remain in the Discussion section.

Discussion

The discussion appropriately interprets the results in context and highlights the clinical importance of standardizing care and reducing antibiotic overuse. It is particularly valuable that professional autonomy was not perceived as a barrier, distinguishing this study from previous literature.

Suggestions:

• Expand on how the tool can complement clinicians' intuition, encouraging buy-in from senior staff.

• Elaborate on applicability in different health systems and suggest strategies to increase "tension for change."

• Consider mentioning potential cost-effectiveness or system-level benefits to further justify implementation.

Conclusion

The conclusion effectively summarizes the study's findings and emphasizes the potential impact on pediatric care.

Suggestions:

• Prioritize key actionable steps, such as EHR integration, CRP accessibility, and user training.

• Add a forward-looking statement regarding next steps (pilot testing, measuring outcomes).

• Briefly mention the potential for application beyond the Dutch setting to enhance international relevance.

Overall Assessment:

This is a well-designed and clinically meaningful study with strong methodological underpinnings. Addressing the above minor suggestions would further clarify the research question, strengthen the practical implications, and make the results more impactful for both local and international readers.

VERSION 1 - AUTHOR RESPONSE

REVIEWER 1

Abstract:

Comment: 'It is not readily apparent what the Feverkidstool is for in the abstract. A short descriptor added to the objectives can help clarify.'

Revision: In response to the reviewer we added that it concerns managing children with fever: ' This study aimed to identify determinants that hinder or facilitate implementation of the Feverkidstool, a clinical decision support tool offering a quantitative, evidence-based approach, to manage children with fever in the ED setting.'

Comment: 'Consider presenting the Results section by CFIR domains.'

Revision: We thank the reviewer for this suggestion and have incorporated the comment into the Results section of the manuscript. However, adding it to the abstract would exceed the word limit and provide limited additional value in that context.

Comment: 'Be careful to not overstate the implications of this present study in the last two sentences.'

Revision: We tuned down the conclusion and rephrased the last two sentences as follows (page 3): ‘This may potentially lead to a more standardized approach and reduce unnecessary antibiotic prescriptions.’

Introduction

Comment: ‘In the first paragraph, the Feverkidstool is briefly described (“… 11 clinical variables, including C-reactive protein.”) As written, I thought other lab values would be included in the tool [in addition to CRP]. However, upon looking up Reference #3, I learned that Feverkidstool does not include any other lab values, but does include history of fever duration, vitals, and exam findings. To provide readers with more thorough understanding of Feverkidstool, consider adding a table/figure or more details to the text.’

Revision: Thanks for this useful suggestion, we have added a figure (figure 1) including the details of the Feverkidstool.

Comment: ‘Thorough Introduction with strong rationale supporting the need for this present study. Study does a good job at connecting the evidence with the need.’

Revision: Thank you for the comment.

Comment: ‘It is unclear what research, if any, has explored implementation of Feverkidstool. Consider explicitly stating if such research has been conducted on specifically the Feverkidstool.’

Revision: We have provided the following information on page 6: ‘Although impact analysis has been performed fort the FKT, no research has yet been performed to specifically examine implementation of the Feverkidstool.’

Methods

Comment: Did the inclusion criteria require a certain amount of experience in the ED (what if a resident has not rotated through the ED yet)?

Revision: Indeed, this was an inclusion criterium, we have specified this in the manuscript: ‘For inclusion we required a minimum of six months of experience as a pediatrician or pediatric resident, including clinical work in the ED.’

Comment: ‘The paper nicely explains that pediatric emergency care is not a subspecialty; however, it remains unclear what other settings, if any, these pediatricians work (i.e., outpatient, inpatient.’

Revision: Thanks for the comment, for this study most important to mention is that pediatrics and pediatric residents work at the ED. To clarify this a bit better we have adjusted the text: ‘Potential end-users of the Feverkidstool were recruited for the interviews. In the Netherlands, pediatric emergency medicine is not formally recognized as a subspecialty; however, all pediatricians and pediatric residents receive training in the assessment of children with emergency conditions (14), and were therefore eligible for recruitment.’ (page 7).

Comment: ‘Analysis: Please provide the reason, if any, for not taking field notes.’

Revision: As all interviews were recorded we did not take field notes in order not to interrupt the interviews. In the text we have added this information: ‘Interviews were audio-recorded and transcribed for analysis. To avoid interrupting the flow of conversation, no field notes were taken during the interviews.’ (page 9).

Comment: ‘Was this study reviewed/approved by an ethics board?’

Revision: In response to the reviewer’s comment, we have added this to the manuscript: ‘Ethical approval for this study was obtained from the Medical Ethical Committee of the Erasmus Medical Center (MEC-2022-0221). Participants provided informed consent prior to participation.’

Results:

Comment: ‘In general, many of the descriptions of the findings lacked depth. Statements were made about the constructs that lacked explanations for “why” and “how.” For example, Intervention characteristics: Did any of the participants elaborate more on why CRP testing would hinder adoption? Or why they would not use the tool for children with comorbidities? (page 13). Another example, Implementation domain: What scientific evidence and data analysis did participants want to have (e.g., clinical outcomes data?)? (page 15)’

Revision: We acknowledge that some results could benefit from additional detail and have

therefore provided further explanation in the Results section. For example, we added: 'Also, some would not use the tool for children with comorbidities as antibiotics are often started more quickly in this group due to a perceived higher risk of serious infection.' (page 15) and 'They believed the tool may be more applicable in cases involving less severely ill children, where uncertainty and differing opinions regarding management are greater, potentially leading to differences in uptake across hospital settings.' (page 15).

Comment: 'It would be helpful to include information throughout the Results section to clarify how common (or ubiquitous) a perception was.'

Revision: We appreciate the reviewer's suggestion and agree that indicating how common certain perceptions were could add useful context. At the same time, we felt that adding detailed frequencies throughout the Results would overload the section. Therefore, we rephrased parts of the Results to include some quantitative indications, such as when a perception was mentioned by all respondents. We also updated Table 2 to show which perceptions were mentioned five times or more. We believe this provides a reasonable balance between offering insight into how often themes were raised and keeping the Results section readable.

Comment: 'Also including details on whether perceptions were largely expressed by a certain provider type (which is provided in the Individual domain but not others).'

Revision: We agree that specifying which provider types expressed certain perceptions is of interest. However, this distinction was only relevant for one other perception namely the usefulness of testing one's own clinical judgment. We have added the following clarification on page 16/17: 'Some physicians mentioned that using it to explain decisions to patients could be valuable, but this was not considered useful by all.'

Comment: 'Including any notable deviants, if any, would be useful.'

Revision: There was a single notable deviant, which we have now included in the manuscript: 'Some physicians mentioned that using it to explain decisions to patients could be valuable, but this was not considered useful by all.'

Comment: ‘Individual domain: The perspectives of more experienced doctors [about the less experienced doctors] is provided. What were the perspectives of the less experienced doctors? Were they more willing to adopt/rely on the tool? (page 14).’

Revision: We have added the perspective of less experienced doctors (page 16): ‘Experienced doctors tend to rely more confidently on their own intuition but believe the tool is valuable for less experienced colleagues, who mentioned relying on their own intuition less frequently.’

Comment: ‘Table 2: Please clarify what the labels are in the last column. Does the “F” for “F2” have any significance? (Similarly, “L” for “L4”? “H” for “H5”?)’

Revision: We have clarified the labeling in Table 2. The letters in the last column now represent the hospitals (A, B, C), and the numbers identify individual respondents within each hospital. In total, eighteen respondents from four hospitals participated, numbered 1 through 18.

Discussion:

Comment: ‘Regarding the first sentence, is this a general implementation strategy or for a specific ED? (page 15)’

Revision: We apologize for the confusion, The phrase ‘will be used for an implementation strategy for the Feverkidstool at the ED’ is more appropriate for the implications of the research findings discussed later. We have moved it to page 21.

Comment: ‘(page 15, lines 40-43) Please provide a citation, if any, supporting the statement about the tool precenting unnecessary diagnostic procedures and antibiotics.’

Revision: We have added a supporting reference for the statement regarding the tool’s potential to prevent unnecessary diagnostic procedures and antibiotic use: ‘The Feverkidstool could help prevent unnecessary diagnostic procedures and reduce the frequent use of antibiotics in cases of uncertainty (5).’

Comment: ‘Paragraphs 2-4 could be rearranged to group together similar concepts (e.g. barriers related to antibiotics and bacterial infections, barriers related to comorbidities, logistical and professional barriers).’

Revision: Thanks for the suggestion, we have revised the discussion section to group related concepts together, as suggested, including barriers concerning antibiotics, comorbidities, and logistical or professional issues.

Comment: ‘There was a note about including nurses in implementation in the results. Given this study emphasizes the need to include key stakeholders in the implementation phase, it would be important to mention again/expand on this finding either in the discussion or in further research about incorporating nurses as key stakeholders.’

Revision: We have incorporated this point in the Discussion section (page 19): ‘Also, including nurses in the implementation process is essential to support full uptake. Because nurses perform the initial assessment, they are well positioned to identify eligible patients and initiate early use of the tool. Their active involvement therefore facilitates integration of the tool into routine practice in a more coordinated, team-based manner.’

REVIEWER 2

Comment: ‘I cannot see any ethics statement in the manuscript. I would expect particpants to provide informed consent to their interviews.’

Revision: We have incorporated this in the method section of the manuscript as follows: ‘Ethical approval for this study was obtained from the Medical Ethical Committee of the Erasmus Medical Center (MEC-2022-0221). Participants provided informed consent prior to participation.’

Comment: ‘The Feverkidstool ideally should be explained more. On reviewing the tool, it does not have much better accuracy than existing tools or clinical gestalt. Suggest to explain the tool in the methods and acknowledge its limitations in the discussion.’

Revision: This was also raised by reviewer one, and we included a figure showing the variables included in the Feverkidstool (figure 1). For a more detailed explanation of the tool,

we refer to the original publication.

Comment: ‘The discussion is well balanced - and indeed some of the respondents comments that any clinical decision tool should aid Gestalt rather than replace it remains important.’

Revision: Thanks for the comment.

Comment: ‘I would encourage the authors to read this lengthy evidence based blog post on clinical decision tools, essentially highlighting the need for it to be compared to Clinical Gestalt. This could take an additional paragraph in the discussion. See: https://first10em.com/clinical-decision-rules/’

Revision: We thank the reviewer for this suggestion, but a discussion on clinical decision rules versus clinical gestalt is beyond the scope of this study, which primarily examines the implementation and user perceptions of the Feverkidstool.

REVIEWER 3

Abstract

Comment: ‘Specify the most critical facilitator and barrier (e.g., EHR integration as a key barrier).’

Revision: We appreciate the reviewer’s comment. Key facilitators and barriers are presented, but we did not identify any single factor as the most important.

Comment: ‘Strengthen the conclusion by highlighting the potential clinical impact, such as expected antibiotic reduction.’

Revision: Although we thought this message was clear in the conclusion, we have rephrased it a bit: ‘This may potentially lead to a more standardized approach and reduce unnecessary antibiotic prescriptions.’

Comment: ‘Include the number of participants (n=18) in the results for context.’

Revision: This has been incorporated in the results.

Introduction

Comment: ‘End with a clear research question, e.g., “This study aims to systematically identify the determinants that hinder or facilitate the implementation of the Feverkidstool in pediatric EDs, guided by the CFIR framework.”. Briefly justify why qualitative methodology is appropriate for exploring implementation challenges.’

Revision: We have clarified the research question and aim: ‘Therefore, this study aims to obtain insights into anticipated enabling or facilitating factors for implementing the Feverkidstool in EDs, as perceived by physicians working in the ED.’

Methods

Comment: ‘Specify the software used for data management/coding (if any) to enhance reproducibility.’

Revision: No specialized software was employed; data was managed and coded using standard tools (e.g. word), which we consider adequate for reproducibility. We also added in the method section that analyses were performed manually: ‘Interviews were audio-recorded and transcribed for analysis manually.’

Comment: ‘Provide the mean or median interview duration in addition to the range.’

Revision: We added this information in the result section: ‘The interviews lasted between 14-45 minutes (median: 24 min; IQR 21-30).’

Comment: ‘Discuss how participants’ prior familiarity with the Feverkidstool was handled analytically, as this may influence perceptions.’

Revision: We have clarified that participants were not involved in the tool’s development

(‘Individuals involved in the scientific development of the tool were excluded from participation.’ page 7) and had therefore not used it before, though some were familiar with it (Table 1). Therefore, we believe no changes to the analysis were required.

Results

Comment: ‘Add a brief summary paragraph or figure highlighting the top three facilitators and top three barriers for quick reference.’

Revision: We appreciate the reviewer’s suggestion. However, the importance of facilitators and barriers cannot be determined solely by frequency; we did not include a summary paragraph or figure. We have, however, provided information on facilitators and barriers mentioned five times or more (see the response to Reviewer 1, comment 12).

Comment: ‘Where feasible, quantify themes (e.g., “12/18 participants mentioned this barrier”) to indicate relative importance.’

Revision: Although we thought qualitative research does not ask for quantifiable results, as noted in our previous response (reviewer 3, comment 8 and reviewer 1, comment 12), we have indicated the number of participants mentioning a perspective five times or more.

Comment: ‘Ensure that interpretive comments and implementation solutions remain in the Discussion section.’

Revision: We have reviewed the placement of all interpretive remarks and implementation suggestions. In the implementation domain subsection of the results, we clarified that the content strictly reflects participants’ views rather than our own interpretations. Additionally, we added the phrase “as reported by end-users” in the first part of the discussion to ensure a clear separation between findings and interpretation.

Discussion

Comment: ‘Expand on how the tool can complement clinicians’ intuition, encouraging buy-in from senior staff.’

Revision: We agree that this is important to add and have therefore added the following sentence: ‘Besides providing advice, the Feverkidstool also contributes to a standardized evaluation of febrile children, which may be more transferable than clinicians’ intuition

across different healthcare providers during supervision or shift transitions.’

Comment: ‘Elaborate on applicability in different health systems and suggest strategies to increase “tension for change.”’.

Revision: We have elaborated more on the healthcare system and added the following sentence (page 20): ‘Also, when implementing the Feverkidstool internationally, stakeholders from countries with different healthcare systems should be questioned (e.g., primary vs. secondary care–led systems) should be consulted to ensure applicability across diverse settings and to identify context-specific barriers and drivers’.

Comment: Consider mentioning potential cost-effectiveness or system-level benefits to further justify implementation.

Revision: We agree that cost-effectiveness and broader system-level benefits are important considerations, although they fall outside the primary scope of the current study. We therefore added a sentence to the Future research section to address this point: ‘Next, as implementation progresses, further research should focus on evaluating the implementation phase, including the actual use of the Feverkidstool, its usability, and the cost- and clinical-effectiveness. After, a postimplementation study can be conducted to evaluate its impact on clinical outcomes in a real clinical setting.’

Additionally, we refer to the cost-effectiveness study by van de Maat et al. (2020), which demonstrated the potential economic value of implementing the Feverkidstool in pediatric emergency care.

Conclusion

Comment: ‘Prioritize key actionable steps, such as EHR integration, CRP accessibility, and user training.'

Revision: We revised the Conclusion section and added the following sentence: ‘Findings indicate that key actionable priorities for implementation include integrating the tool into the EHR and providing evidence-based education, which should be central components of the implementation strategy.’ We did not include CRP accessibility, as this was not identified as a key actionable step and therefore was not prioritized.

Comment: Add a forward-looking statement regarding next steps (pilot testing, measuring outcomes). Briefly mention the potential for application beyond the Dutch setting to enhance international relevance.

Revision: We have rephrased our conclusion and added the following sentence: ‘Next steps involve pilot testing and evaluating usability, adoption, and clinical impact. Additionally, exploring implementation in different international healthcare systems will be important to adapt strategies to local contexts.’

VERSION 2 - REVIEW

Reviewer **2**

Name **Keijzers, Gerben**

Affiliation **Gold Coast Hospital and Health Service, School of Medicine**

Date **09-Dec-2025**

COI

Thanks for thoughtfully addressing or appropriately rebutting the reviewers' comments.

One of my original comments was rebutted for being 'out of scope'.

I suggested that any research on clinical decision tools should inlcude a comparison with clinical judgment/gestalt.

In my view this is clearly within scope, as you are assessing barriers to implement a CDT (Feverkidstool), but really clinicians should only implement this tool, if it has been shown to outperform clinical judgment in a head to head comparison. The lengthy reference I included in the original review is very much worth reflecting over if this will lead to further research on clinical decsion tools.

The reality is that the published research on the Feverkidstool does NOT suggests any other tools or clinical judgment, and therefore a few sentences in the Discussion to acknowledge this and to highlight the importance of clinical context remains important.

Readers will also value a link back to the importance of clinical experience and gestalt (as most clinical decision tools are derived from experience and gestalt)

Reviewer **3**

Name **Kaya, Murtaza**

Affiliation **Kutahya Saglik Bilimleri Universitesi, Emergency Department**

Date **10-Dec-2025**

COI

Dear Editor,

I would like to thank the authors for their thorough and constructive revision of the manuscript. I am pleased to note that the key points highlighted in my initial review have been carefully addressed. In particular, the abstract has been strengthened with clearer emphasis on primary facilitators and barriers, methodological details have been clarified, and the Results and Discussion sections now provide a more structured and comprehensive interpretation aligned with the CFIR framework.

The authors have successfully incorporated contextual details such as participant familiarity, median interview duration, and quantification of themes where appropriate. Additionally, the enhanced discussion on clinical applicability, implementation challenges, and future research directions substantially improves the manuscript's overall clarity and relevance.

Readers will also value a link back to the importance of transparent, and clinically meaningful paper. In its current form, the manuscript meets the standards for publication and provides valuable insight into implementation factors for clinical decision tools in pediatric emergency care.

I believe the manuscript is now suitable for acceptance.

VERSION 2 - AUTHOR RESPONSE

Comment:

'One of my original comments was rebutted for being 'out of scope'.

I suggested that any research on clinical decision tools should inlcude a comparison with clinical judgment/gestalt.

In my view this is clearly within scope, as you are assessing barriers to implement a CDT (Feverkidstool), but really clinicians should only implement this tool, if it has been shown to outperform clinical judgment in a head to head comparison. The lengthy reference I included in the original review is very much worth reflecting over if this will lead to further research on clinical decsion tools.

The reality is that the published research on the Feverkidstool does NOT suggests any other tools or clinical judgment, and therefore a few sentences in the Discussion to acknowledge this and to highlight the importance of clinical context remains important.

Readers will also value a link back to the importance of clinical experience and gestalt (as most clinical decision tools are derived from experience and gestalt)'

Revision:

We thank the reviewer for revisiting this point. In our initial revision we considered the suggestion of including reflection on clinical judgment and gestalt to be out of scope. After careful reconsideration, we agree that acknowledging the role of clinical judgment and clinician expertise is relevant and valuable in the context of tool adoption and implementation of decisions. We therefore revised the Discussion accordingly to better reflect this perspective and to reinforce the importance of clinical context when interpreting tool recommendations.

We acknowledge that a direct performance comparison between the Feverkidstool and clinician gestalt has not yet been conducted. Rather than evaluating superiority over clinical judgment at this stage, the FKT is intended to be used by clinicians alongside their own diagnostic reasoning. More broadly, this reflects the role of CDSTs in general, where tools are meant to structure assessment, reduce unwarranted variation, and strengthen (but not replace) clinical expertise, particularly in moments of diagnostic uncertainty. This perspective is now explicitly stated in the discussion, leading into our clarification that: "In addition, the FKT provides a single-point assessment with immediate advice, whereas infectious conditions can evolve over time, progressing from subtle early signs to clear management indications. End users particularly valued the immediate advice of the tool in less clear, early phase presentations with highest diagnostic uncertainty (see quotations in the construct 'relative advantage' in table 2). This aligns with the way the Feverkidstool, and other CDST should be used by complementing rather than replacing physicians' clinical judgment, and that recommendations should always be interpreted within the broader clinical context of the individual patient. The FKT has been validated for single-moment assessment only, and its use beyond this context has not been formally evaluated.'' on page 19. And also, in our concluding paragraph on page 20/21: 'Next steps involve pilot testing and evaluating usability, adoption, and clinical impact, acknowledging that the Feverkidstool is intended to support clinical decision-making by complementing physicians' clinical judgment within the broader clinical context.'

Also, we added further emphasis on the importance of real-world evaluation, including post-implementation studies, to determine the true added value of the Feverkidstool when used alongside clinician judgment. This is reflected on page 21 with our addition that: “Such evaluation is essential, as the added value of clinical decision tools can only be determined when they are used in routine practice and assessed in relation to real-world clinical decision-making.”
